# P18 You can’t prescribe what you don’t have: the limitations of addressing the management of symptomatic sexually transmitted infections in a rural Zambian health post

**DOI:** 10.1093/jacamr/dlad143.022

**Published:** 2024-01-03

**Authors:** Olivia Duffy, Eloise Smellie, Christone Silungwe

**Affiliations:** On Call Africa, UK; On Call Africa, UK; On Call Africa, Zambia

## Abstract

**Background:**

Management of sexually transmitted infections (STIs) remains a significant global issue, with more than one million cases acquired each day worldwide.^1^ Growing antimicrobial resistance poses a significant threat to addressing this global burden of disease. While STIs are a recognized public health problem in Zambia,^2^ resistance remains largely an unknown entity.^3^ This is particularly true of rural areas where there is no local resistance data available and very few microbiology labs. In this setting, the WHO syndromic approach to STI management is recommended.^4^

**Objectives:**

To reduce inappropriate antimicrobial prescribing for genital presentations in a rural health post in Zambia by the provision of educational resources and collaborative teaching based on the WHO syndromic approach to STIs. Volunteers for a non-governmental organization designed and implemented a clinical audit to improve diagnosis and management of genital symptoms in a rural Zambian health post as part of an ongoing antimicrobial stewardship programme.^5^

**Methods:**

Antibiotic use was measured at two intervals over a 1 year period (April to June 2022 and January to March 2023). Patient records were reviewed against an inclusion criterion of a relevant genitourinary diagnosis. The data was analysed for diagnostic accuracy and for the appropriateness of antimicrobial prescribing using the appropriate prescribing ratio (APR).^6,7^ National guidelines and WHO recommended alternatives were used as the standard. Data collection and analysis was approved by the Zambian Ministry of Health. The WHO granted permission to use and adapt its materials (ID:202300108). The intervention consisted of collaborative teaching structured around the implementation of simple flow charts based on the WHO syndromic approach and national Zambian guidelines. One-to-one mentoring was delivered over a period of three weeks.

**Results:**

The proportion of plausible differentials increased from 52.5% (*n*=40) to 80.5% (*n*=41). APR increased from 5% to 9.8%. The proportion of inadequate and unnecessary prescriptions reduced, falling from 85% to 73.2% and 10% to 2.4%, respectively. Suboptimal prescriptions increased, rising from 0% to 14.6%.

**Conclusions:**

This audit supports the use of guidelines and education based around the WHO syndromic approach to improve the diagnosis of genital infections in low resource settings. The limited improvement in prescribing was most likely influenced by the severe antibiotic shortages reported by local healthcare workers.^5^ This highlights the significance of addressing supply chains and ordering processes, particularly to rural areas. It is likely that improvements in appropriate prescribing rates will depend on parallel improvements in local antimicrobial availability. No educational intervention can overcome an empty drug cupboard, but education can be used to conserve the limited stock through more selective use. Improved access to either laboratories or point of care testing would make the decision to prescribe easier. However, in their absence, increasing adherence to the WHO syndromic approach has the potential to limit unnecessary prescriptions in low-resource rural settings, improving resource conservation and antimicrobial stewardship.
